# In depth investigation of the metabolism of *Nectandra megapotamica* chemotypes

**DOI:** 10.1371/journal.pone.0201996

**Published:** 2018-08-06

**Authors:** Katyuce de S. Farias, Thierry Delatte, Rosani do C. de O. Arruda, Flavio M. Alves, Denise B. Silva, Jules Beekwilder, Carlos A. Carollo

**Affiliations:** 1 Laboratório de Produtos Naturais e Espectrometria de Massas, Faculdade de Ciências Farmacêuticas, Alimentos e Nutrição, Universidade Federal de Mato Grosso do Sul, Campo Grande, MS, Brasil; 2 Laboratory of Plant Physiology, Wageningen University, Wageningen, PB, The Netherlands; 3 Wageningen Plant Research, Wageningen, The Netherlands; 4 Laboratório de Anatomia Vegetal, Universidade Federal de Mato Grosso do Sul, Campo Grande, MS, Brasil; 5 Laboratório de Botânica, Universidade Federal de Mato Grosso do Sul, Campo Grande, MS, Brasil; College of Agricultural Sciences, UNITED STATES

## Abstract

Plants produce a wide range of secondary metabolites. Within a single species, chemotypes can be distinguished by the differences in the composition of the secondary metabolites. Herein, we evaluated *Nectandra megapotamica* (Spreng.) chemotypes and the balance of different classes of metabolites to verify how significant differences in plant metabolism are regarding chemotypes. We collected *N*. *megapotamica* leaves from eight adult plants in two Brazilian states. The essential oils and ethanol/water extracts were analyzed by GC-MS and LC-DAD-MS, respectively. Histochemical tests were performed, as well as chemical analyses of leaves from adaxial and abaxial foliar surfaces of *N*. *megapotamica*, and the stereochemistry of α-bisabolol was determined. Two different chemotypes, based on volatile compounds, were identified, distinguished by the presence of isospathulenol, α-bisabolol, β-bisabolene, and (E)-nerolidol for chemotype A, and bicyclogermacrene and elemicin for chemotype B. A stereochemical analysis of chemotype A extract revealed (+)-α-bisabolol enantiomer. Histochemical tests of chemotypes showed similar results and suggested the presence of essential oil in idioblasts stained with the dye NADI. The analyses of chemotype A leaves by GC-MS revealed similar compositions for abaxial and adaxial surfaces, such pattern was also observed for chemotype B. Medium and high polarity metabolites showed high chemical similarities between the chemotypes, highlighting the presence of proanthocyanidins and glycosylated flavonoids (*O*- and *C*-glycosides). Thus, *N*. *megapotamica* produced distinct volatile chemotypes with highly conserved medium to high polarity compounds. Such results suggest that phenolic derivatives have a basal physiological function, while genetic or environmental differences lead to differentiation in volatile profiles of *N*. *megapotamica*.

## Introduction

Plants can produce a variety of compounds, with different structural arrangements [1], and, based on these criteria, some specimens are differentiated by chemotypes, which are related to chemical polymorphism within species [2]. There are several factors that explain polymorphism, including differences in ecological environment and genetic mutations [3].

Chemotypes significantly impact the economic value of plants, as they affect the biological effect, yield, and quality of plant material, due to the differentiation in chemical composition. A study about *Murraya koenigii* (L.) Spreng. (Rutaceae), also known as curry tree, presented two chemotypes, chemotype A, rich in pinene and sabinene, and chemotype B, which does not accumulate sabinene. The authors noted that sabinene contributes to aroma and flavor and highlighted that pinene and sabinene are valuable to the flavor industry, therefore, chemotype A is ideal for commercialization [4].

Furthermore, despite the importance of alterations observed in chemotypes, currently, ecological effects are poorly understood. Wallis et al. [5] emphasize that the presence of chemotypes (found in *Eucalyptus globulus*) suggest an evolutionary advantage for the species, increasing the variability of populations, which improves vegetative defense against herbivory. Moreover, Karban et al. [6] showed plants that produce the same volatile chemotype have more effective communication than plants with different composition, and such plants can coordinate their physiological processes to decrease herbivore attack.

Studies on the chemical variability of Lauraceae species have indicated the occurrence of chemotypes in this family. For example, *Cinnamomum osmophloeum* Kaneh has five characterized chemotypes and shows larvicidal activities for only two chemotypes (cinnamaldehyde and cinnamaldehyde/cinnamyl acetate) [7]. *Licaria salicijdia* (Sw.) Kosterm, another Lauraceae species, presented high essential oil diversity, and the soil significantly influenced the production of the different chemotypes observed [8].

The Lauraceae species used in the present work, *Nectandra megapotamica* (Spreng.), belongs to the genus *Nectandra* rol. Ex rottb., which is the second largest Lauraceae family with about 114 species [9]. This plant is commonly known as “canela preta,” and used in folk medicine to treat fungal infections, diarrhea, pain, and fever [10]. *N*. *megapotamica* is found in different Brazilian regions, as well as in other South American countries [11]. Secondary metabolites have been identified in *N*. *megapotamica*, including alkaloids [12], phenylpropanoids [13], and tetrahydrofuran lignans [14].

Two studies performed on *N*. *megapotamica* from different places of Brazil (Midwest and South East) showed a possible chemotype for this species. Romoff et al. [15] identified high levels of sesquiterpene (α-bisabolol) in essential oils extracted from the leaves (62.3–69.4%, relative percentage), while Amaral et al. [16] identified different main compounds in the leaves (α-pinene and bicyclogermacrene) and an absence of α-bisabolol.

An interesting finding was reported by Nehme et al. [17] for *C*. *mandioccana*, which showed chemotypes for styrylpyrone derivatives, combined with a conserved profile of flavonoid glycosides. Few studies have focused on chemotype differences at the level of plant metabolism for different compound classes, with no prior research about other Lauraceae species. Herein, we investigated if *N*. *megapotamica* chemotypes presented the same volatile and phenolic compounds.

## Materials and methods

### Ethics statement

This study did not involve any endangered species or from Brazilian legally protected areas. Therefore, the owners previously authorized the collect in particular areas, while it was not necessary to legal authorization when collected in urban areas of landscape planning.

### Acquisition of plant material

*N*. *megapotamica* leaves were collected from eight adult plants in two Brazilian states (Mato Grosso do Sul and São Paulo), as well as two *N*. *megapotamica* seedlings. Flávio Macedo Alves identified the species, and the vouchers were deposited into the Herbarium at the Federal University of Mato Grosso do Sul (CGMS), Mato Grosso do Sul, Brazil, and the Herbarium of Florestal Institute of São Paulo (SPSF), São Paulo, Brazil. The data from the collection is presented in Table 1, and vouchers and complement information are in S1 Table.

**Table 1:** Identification and location of studied vegetal materials.

Abbreviation	Materials	Location city-state-country
C1	*N*. *megapotamica*	Campo Grande—MS, Brazil (C)
C2	*N*. *megapotamica*	Campo Grande—MS, Brazil (C)
C3	*N*. *megapotamica*	Campo Grande—MS, Brazil (C)
M1	*N*. *megapotamica*	Maracaju—MS, Brazil (M)
P1	*N*. *megapotamica*	Ponta Porã - MS, Brazil (P)
P2	*N*. *megapotamica*	Ponta Porã - MS, Brazil (P)
S1	*N*. *megapotamica*	São Paulo—SP, Brazil (S)
S2	*N*. *megapotamica*	São Paulo—SP, Brazil (S)
S3	*N*. *megapotamica*	São Paulo—SP, Brazil (S)
S4	*N*. *megapotamica*	São Paulo—SP, Brazil (S)
S5	*N*. *megapotamica*	São Paulo—SP, Brazil (S)
S6	*N*. *megapotamica—*seedling	São Paulo—SP, Brazil (S)
S7	*N*. *megapotamica—*seedling	São Paulo—SP, Brazil (S)

### Preparation of plant material

Fresh leaves from C1, C2 and C3 (C region), M1 (M region), P1 and P2 (P region), and S1, S2, S3, S4 and S5 (S region) were weighed (approximately 125 g) and milled, and their essential oils were extracted by hydrodistillation in a Clevenger apparatus for 5 hours.

The yields of essential oils were: C1 (0.96%), C2 (0.79%), C3 (0.17%), M1 (0.26%), P1 (0.24%), P2 (0.34%), S1 (0.99%), S2 (0.56%), S3 (0.99%), S4 (0.67%) and S5 (0.55%).

### Gas chromatography-mass spectrometry (GC-MS)

The essential oils from C1, C2, C3, M1, P1, P2, S6 and S7 were analyzed using a Shimadzu QP2010 gas chromatograph equipped with COA-20i auto-injector, RTx-5MS capillary column (30 m x 0.25 mm x 0.25 μm), at the concentration of 1 mg/mL in split mode (1:5). The carrier gas used was helium.

The injection temperature and interface used was 250°C, and the program for C1, C2, C3, M1, P1, P2 followed the gradient: from 0 to 6 minutes isothermal method at 75°C, from 6.1 to 11 min heating ramp of 20°C/min to 170°C, from 11.1 min to 16 min, the isothermal method at 170°C, from 16.1 min to 34 min, 5°C/min heating to 260°C, and finally from 34.1 to 48.75 min isothermal method at 260° C.

The samples S6 and S7 were analyzed in 60 min using the isothermal method, with an initial temperature of 60°C and final of 240°C. The mass spectra were obtained by electron ionization at 70 eV.

The essential oils of S1, S2, S3, S4, and S5 were analyzed at the natural and synthetic products research center (NPPNS) at the University of São Paulo (USP) using a Shimadzu gas chromatograph, model QP-2010, column EN-5MS (30 m x 0.25 mm x 0.25 μm) at concentration of 1 mg/mL. The split mode ratio was 1:5, and injector and interface temperature was 240°C. The carrier gas used was helium, and the temperature program was an isothermal mode with an initial temperature of 60°C using a heater of 3°C/min up to 240°C.

The mass spectra were obtained through electron ionization at 70 eV in all the analyses. Retention indices were calculated using C8-C26 alkane standards (Sigma Aldrich). To interpretate the spectra we used the NIST, WILEY, FFNSC libraries and compared retention indices with the literature [18].

For chiral analysis, we prepared dichloromethane extract of sample S4, commercial samples (*Eremanthus erythropappus* ((-)-α-bisabolol), *Salvia stenophylla* ((-)-α-bisabolol and (+)-epi-α-bisabolol), *Lippia dulcis* ((+/-)-epi-α-bisabolol) and standards (+/-)-α-bisabolol. The racemic bisabolol (Dragostanol) standard was kindly provided by Dr. Matthias Panten from Symrise.

The leaf extracts from S4 and commercial samples were prepared through sonication for 10 minutes with dichloromethane, followed by centrifugation at 1200 rpm, collection of the supernatants, removal of water residues using Na_2_SO_4,_ and GC-MS analysis. The assay was performed according to König and Hochmuth [19] with minor modifications.

The gas chromatograph 7890A (Agilent) was coupled to MSD 5975C (Agilent), and the monitored mass range was *m/z* 45–300. The injection temperature was 250°C in split mode (9:1) using the enantioselective column Alpha DEXtm 120 (Supelco; Bellefonte, PA) with 30m x 0.25mm x 0.25μm and ZB-5MS column (Phenomenex; USA). The initial temperature was 50°C for 1 min, followed by heater of 2°C/min up to 170°C.

### Processing of data obtained by GC-MS

The mass signals of raw data files from GC-MS analyses were extracted and aligned by metAlign software [20], producing 3222 entrances. Subsequently, the data was reduced by MSClust software [21], resulting in 79 reconstructed metabolites. Finally, the processed data was statistically analysed by Metaboanalyst 4.0 platform [22] using OrthoPLSDA. For OrthoPLSDA, the data was normalized by median and log average transformations.

### Histochemical analysis

We cut cross sections of *N*. *megapotamica* leaves by hand. The material was stained with NADI reagent (naphthol and N-Diethyl-*p*-phenylenediamine), ferric chloride, Sudan IV, and Nile blue sulfate [23]. The images were captured under microscope Leica Microsystems with 100x, 200x, 400x and 1000x magnification.

To count idioblasts on the adaxial and abaxial leaf surfaces, we used five *N*. *megapotamica* leaves at similar development stages, which were located 3 cm above the petiole with the surface size of 2x2 cm. The measurements were performed using 20 microscopic fields at a magnification of 200 diameters, and the numbers are presented as mean and standard error of the means. A t-test was used for statistical analyses.

The fresh and healthy adult leaves from S6 and S7 were prepared as described above. Afterwards, the adaxial and abaxial foliar surfaces were scraped to obtain the outermost cells. The material was placed in a 2 mL vial to extract volatile compounds by solid-phase microextraction (SPME) and analyses by GC-MS. The polydimethylsiloxane fiber (Supelco, Bellafonte, PA, USA) (thickness 100 μm) attached to SPME Holder (57330-U) was exposed to the sample for 50 minutes at 40°C for extraction in the vials. The desorption of volatile compounds was carried out through fiber exposure directly in the gas chromatograph injection chamber at 250°C for 4 minutes.

### Liquid chromatography coupled to diode-array detection and mass spectrometry (LC-DAD-EM)

Fresh *N*. *megapotamica* leaves were pulverized in an analytical mill, tamized (mesh 24), and 50 mg were extracted with methanol/water 7:3 (v/v) added 1% acetic acid in ultrasound bath for 10 minutes, and centrifuged at 3000 RPM for 10 minutes. The supernatants were collected, filtered (0.22 μM pore, membrane PTFE, Millex^®^ filters), and analyzed on LC-DAD-MS.

The analyses were performed on an UFLC (LC-20AD, Shimadzu) system coupled to a diode array detector (DAD) and a mass spectrometer (ESI-qTOF microTOF-Q III, Bruker Daltonics). A chromatography column Kinetex C-18 (2.6 μ, 150 x 2.2 mm, Phenomenex) was used with a pre-column of the same stationary phase material.

The mobile phase was composed of ultrapure water (Phase A) and acetonitrile (Phase B), both added 0.1% of formic acid (v,v). The gradient elution profile was as follows: 0–2 min- 3% of B; 2.1–25 min- 3–25% of B; 25.1–40 min- 25–80% of B followed by column washing and reconditioning (8 minutes).

The column temperature was 50°C, the flow rate was 0.3 mL/min using 2 μL for the injection volume. The mass spectrometry analyses were obtained in positive and negative ion mode (*m/z* 120–1200), and the compounds were monitored at wavelengths from 240 to 800 nm. The compounds were identified by UV spectral data, accurate mass, and fragmentation profile compared to data from the literature.

## Results and discussion

### Analyses of essential oils by GC-MS

The essential oils obtained from *N*. *megapotamica* in the states of São Paulo (S) and Mato Grosso do Sul (C, M, and P) were analyzed by GC-MS (geographic distribution map–S1 Fig, Supplementary Material). The compounds identified were phenylpropanoids (up to 52.7%), monoterpene hydrocarbons (up to 19.9%), sesquiterpene hydrocarbons (up to 92.3%), oxygenated sesquiterpene (up to 94.5%) and others (up to 4.5%). The compounds are described in Table 2.

**Table 2:** Main components identified by GC-MS from essential oils of *Nectandra megapotamica*.

	Compounds			S1	S2	S3	S4	S5	C1	C2	C3	M1	P1	P2
**n.**	**Monoterpene hydrocarbons**	RI	RIL											
1	limonene	1042	1029	—	—	—	—	—	—	1.3	0.5	—	—	—
2	α-phellandrene	1012	1003	—	—	—	—	—	—	1	—	—	—	—
3	α-pinene	938	939	—	5.2	1.2	—	—	—	3	5.7	—	—	—
4	α-terpinolene	1093	1089	—	—	—	—	—	—	1.6	—	—	—	—
5	β-myrcene	993	991	—	—	—	—	—	—	1.2	—	—	—	—
6	β-pinene	982	979	—	4	—	—	—	—	—	2.2	—	—	—
7	δ-3-carene	1016	1031	—	—	1.6	—	—	—	10.9	—	—	—	—
8	(*E*)-β-ocimene	1060	1050	—	—	—	—	—	—	0.9	0.5	—	—	—
9	(*Z*)-β-ocimene	1011	1037	—	3.3	—	1.5	2.1	—	—	—	—	—	—
	**Total:**			**0**	**12.5**	**2.8**	**1.5**	**2.1**	**0**	**19.9**	**8.9**	**0**	**0**	**0**
	**Hydrocarbon sesquiterpenes**													
10	alloaromadendrene	1475	1486	—	—	—	—	—	1	—	2.6	—	—	—
11	Aromadendrene	1462	1441	—	—	—	—	—	—	1.1	—	—	—	—
12	Bicycloelemene	1351	1336	—	—	—	—	—	—	1.1	0.5	—	—	—
**13**	**bicyclogermacrene**	1520	1500	—	—	—	—	—	5.6	24.8	8.9	**66.7**	28.2	26.3
14	germacrene B	1584	1561	—	—	—	—	—	—	—	1.9	—	—	—
15	germacrene D	1492	1485	—	—	4	1.4	1.2	4.9	—	4.6	18.2	—	4.8
16	α-copaene	1394	1377	—	—	—	—	—	1.4	—	0.3	—	—	—
17	α-curcumene	1479	1475	—	—	—	—	1.2	—	—	—	—	—	—
18	α-guaiene	1431	1440	—	—	—	—	—	—	tr	tr	—	—	—
19	α-muurolene	1500	1500	—	—	—	—	—	3.6	—	—	—	—	—
20	α-santalene	1435	1418	—	—	—	—	—	11.8	—	—	—	—	—
21	β-bergamotene	1454	1436	—	—	—	—	19	—	—	—	—	—	—
22	β-bisabolene	1509	1506	1	13.3	2.5	0.9	1.2	—	—	—	—	—	—
23	β-caryophyllene	1442	1467	—	—	—	—	—	1.5	—	7	4	—	—
24	β-elemene	1401	1391	1.8	3.1	—	—	—	2.2	1	0.9	3.4	3.5	—
25	β-farnesene	1453	1460	—	—	1.6	0.5	—	—	—	—	—	—	—
26	β-santalene	1462	1460	—	—	—	—	1	6.9	—	—	—	—	—
27	β-selinene	1478	1490	—	—	—	—	—	—	0.8	1	—	—	—
28	β-sesquiphellandrene	1506	1523	—	6.7	—	—	32	—	—	—	—	—	—
29	δ-cadinene	1540	1523	—	—	—	—	—	3.8	—	1.1	—	—	—
**30**	**δ-elemene**	1343	1338	13.8	23.8	2.1	—	—	15.6	—	—	—	**32.2**	**37.9**
31	γ-gurjunene	1499	1477	—	—	—	—	—	—	—	—	—	5.3	—
32	*trans*-caryophyllene	1442	1419	—	—	—	—	—	—	8.3	—	—	—	—
33	*trans*-α-bergamotene	1431	1435	—	—	—	—	1.5	4.6	—	—	—	—	—
34	9-epi-(E)-caryophyllene	1461	1466	—	—	—	—	—	2.7	—	—	—	6	—
35	(E)-α-bisabolene	1543	1540	—	—	—	—	1.8	—	—	—	—	—	—
36	(E)-γ-bisabolene	—	—	—	—	—	—	—	—	—	—	—	—	6.4
	**Total**			**16.6**	**46.9**	**10.2**	**2.8**	**58.9**	**65.6**	**37.1**	**28.8**	**92.3**	**75.2**	**75.4**
	**Oxygenated sesquiterpenes**													
37	Elemol	1547	1550	0.6	—	—	—	—	—	—	—	—	—	—
38	Intermedeol	1674	1667	—	—	—	—	—	—	—	0.6	—	—	—
39	Isospathulenol	1636	1619	11.3	26.8	—	—	—	3.8	—	—	—	3	—
40	Spathulenol	1575	1578	1.0	3.2	—	—	—	—	—	—	—	—	—
41	Viridiflorol	1596	1593	2.0	—	—	—	2.7	—	—	0.4	—	—	—
**42**	**α-bisabolol**	**1683**	**1686**	59.7	1.6	84.3	**93.7**	8.9	—	—	—	—	—	—
43	α-bisabolol oxide	1652	1658	1.1	—	—	—	—	—	—	—	—	—	—
44	β-bisabolol	1667	1675	—	—	—	—	3.3	—	—	—	—	—	—
45	τ-cadinol	1624	1640	0.5	2.9	—	—	—	—	—	—	—	—	—
46	(E)-nerolidol	1554	1563	0.6	—	0.9	0.8	6.1	—	—	—	—	—	—
47	(Z)-nerolidol	1552	1533	—	—	—	—	4.7	—	—	—	—	—	—
	**Total**			**76.8**	**34.5**	**85.2**	**94.5**	**25.7**	**3.8**	**0**	**1**	**0**	**3**	**0**
	**Phenylpropanoids**													
**48**	**Elemicin**	1562	1557	—	—	—	—	—	—	**35.9**	**52.7**	5.6	—	—
**49**	**(E)-asarone**	1688	1676	—	—	—	—	—	**22.6**	—	—	—	10.3	15
50	(Z)-asarone	1619	1617	—	—	—	—	—	2.2	1	—	—	—	—
	** Total**			**0**	**0**	**0**	**0**	**0**	**24.8**	**36.9**	**52.7**	**5.6**	**10.3**	**15**
	**Others**													
51	2-ethyl-1-hexanol	1039	1030	—	—	—	—	—	—	—	—	—	3.4	4.5
52	Unknown 1	1527	-	—	—	—	—	0.9	—	3.2	4.1	—	—	—
53	Unknown 2	1684	-	—	—	—	—	2.4	—	—	—	—	—	—
	**Total**			**0**	**0**	**0**	**0**	**3.3**	**0**	**3.2**	**4.1**	**0**	**3.4**	**4.5**
	**% Total Identified**			**93.4**	**93.9**	**98.2**	**98.8**	**90.0**	**94.2**	**97.1**	**95.5**	**97.9**	**91.9**	**94.9**

All individuals were identified as *Nectandra megapotamica*, but from different regions. From the state of Mato Grosso do Sul: C1 to C3: city of Campo Grande; M1: city of Maracaju; P1 and P2: city of Ponta Porã. State of São Paulo: S1 to S5: city of São Paulo. n: number; RI: retention index of the compound; RIL: retention index of literature. The major compounds of each species are in bold.

A total of 53 compounds were identified. The highest relative percentages of compounds were the phenylpropanoids (*E*)-asarone (22.6% in C1), elemicin (35.9% and 52.7% in C2 and C3, respectively), the sesquiterpenes hydrocarbons bicyclogermacrene (66.7% in M1) and δ-elemene with 32.2% in P1 and 37.9% in P2, as well as the oxygenated sesquiterpene α-bisabolol, with 93.7% in the S region.

**Fig 1:**
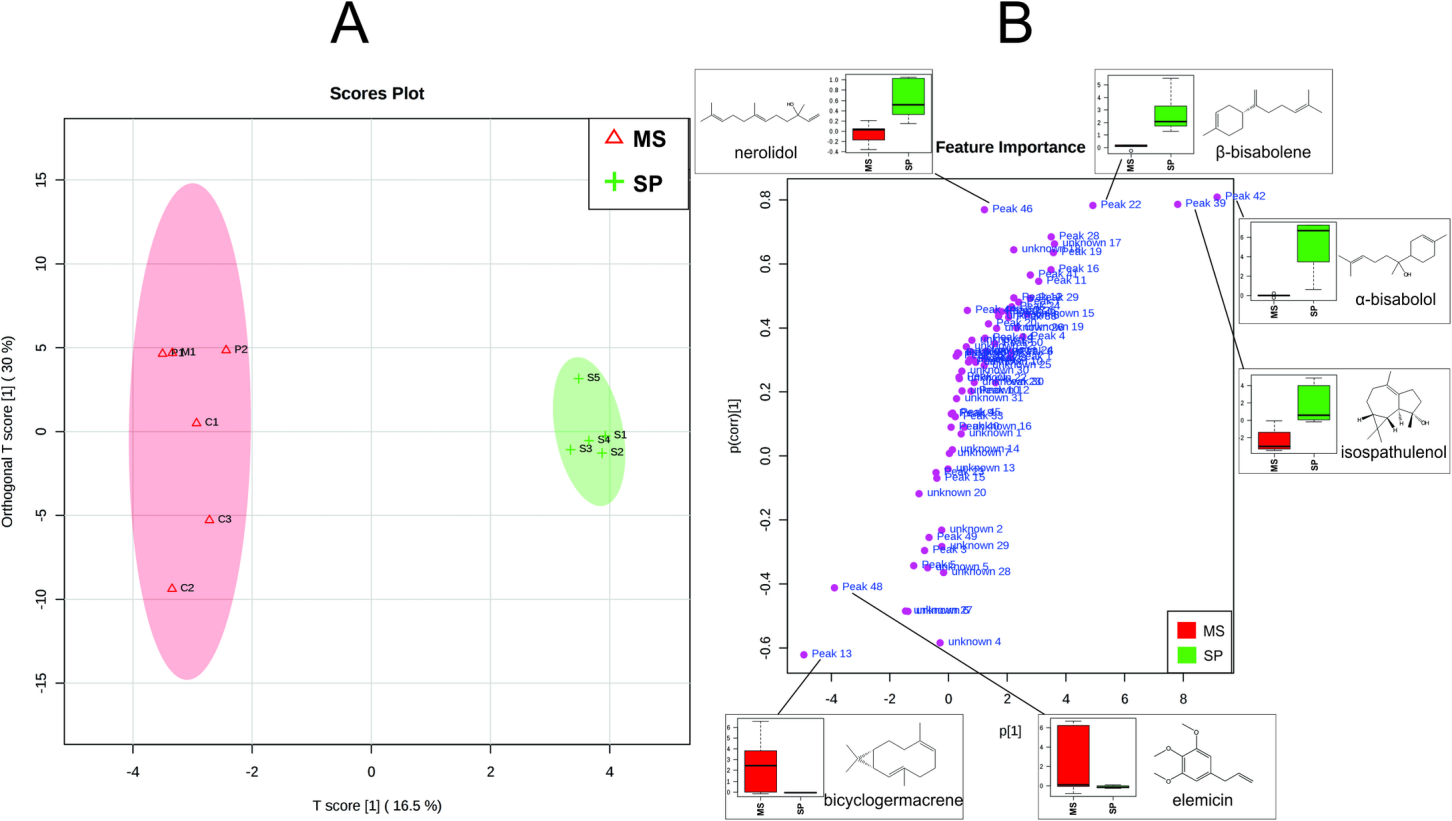
**(A) OrthoPLSDA and (B) Loading of *Nectandra megapotamica***. (SP: Includes individuals from S1 to S5 (state of São Paulo, Brazil). MS: Includes individuals from cities C1 to C3 (Campo Grande, Brazil), M1 (Maracajú, Brazil) and P1 to P2 (Ponta Porã, Brazil) from the state of Mato Grosso do Sul.

To determine the metabolic diversity of the different samples, we displayed samples with an OrthoPLSDA (Fig 1), and observed a clear separation based on the geographic distribution of the samples. From such clear separation, we confirmed two chemotypes for *N*. *megapotamica*.

The chemotype A (from São Paulo state samples) is characterized by the presence of isospathulenol (peak 39), α-bisabolol (peak 42), β-bisabolene (peak 22) and (*E*)-nerolidol (peak 46), while the chemotype B (from Mato Grosso do Sul state samples) by the compounds bicyclogermacrene (peak 13) and elemicin (peak 48).

The chemotype A was most significant for the separation of S region samples and can accumulate more oxygenated sesquiterpenes. On the other hand, the chemotype B was most significant in the separation of samples from the C, M and P regions, which were collected from the same Brazilian state and characterized by the presence of non-oxygenated sesquiterpenes and a phenylpropanoid.

Interestingly, isospathulenol (peak 39), which presented a significant concentration of chemotype A, was not previously reported in the literature for this species, and correlated to the evaluation of volatile chemotypes in *N*. *megapotamica* from different geographic locations. Volatile chemotypes have already been described for species of Lauraceae, as in *Licaria salicijdia* (Sw.) Kosterm [8] and *Cinnumomum zaubatii* [24].

Several factors can affect the chemical composition of specimens, such as circadian rhythm, plant development phase, seasonality, adaptation mechanisms, locality, soil, temperature, water and nutrient availability, biotic pressure, altitude, and ultraviolet radiation [25, 26]. Furthermore, the type of secretory anatomical structures, as well as genetic factors, can also induce chemical variations [27].

However, genetic factors can best explain the quantitative variations of α-bisabolol (peak 42) observed in samples from the São Paulo region since these samples were collected at the same time and geographical location.

Identifying chemotypes can help clarify the distribution and evolution of this species [17], as well as determine economically important species due to high prevalences and incidence of compounds, such as sesquiterpene α-bisabolol (approximately 93.7%), in specific regions. This sesquiterpene was only observed in the specimens from the São Paulo region, and is used for biotechnological purposes, as the essential oil from *Cordia verbenacea*, which is used to produce a phytotherapeutic topical anti-inflammatory called Acheflan^®^ due to its active compound α-humulene (5 mg of the essential oil is equivalent to 0.130 mg of alpha-humulene in cream formulation).

### Histochemical analysis

**Fig 2:**
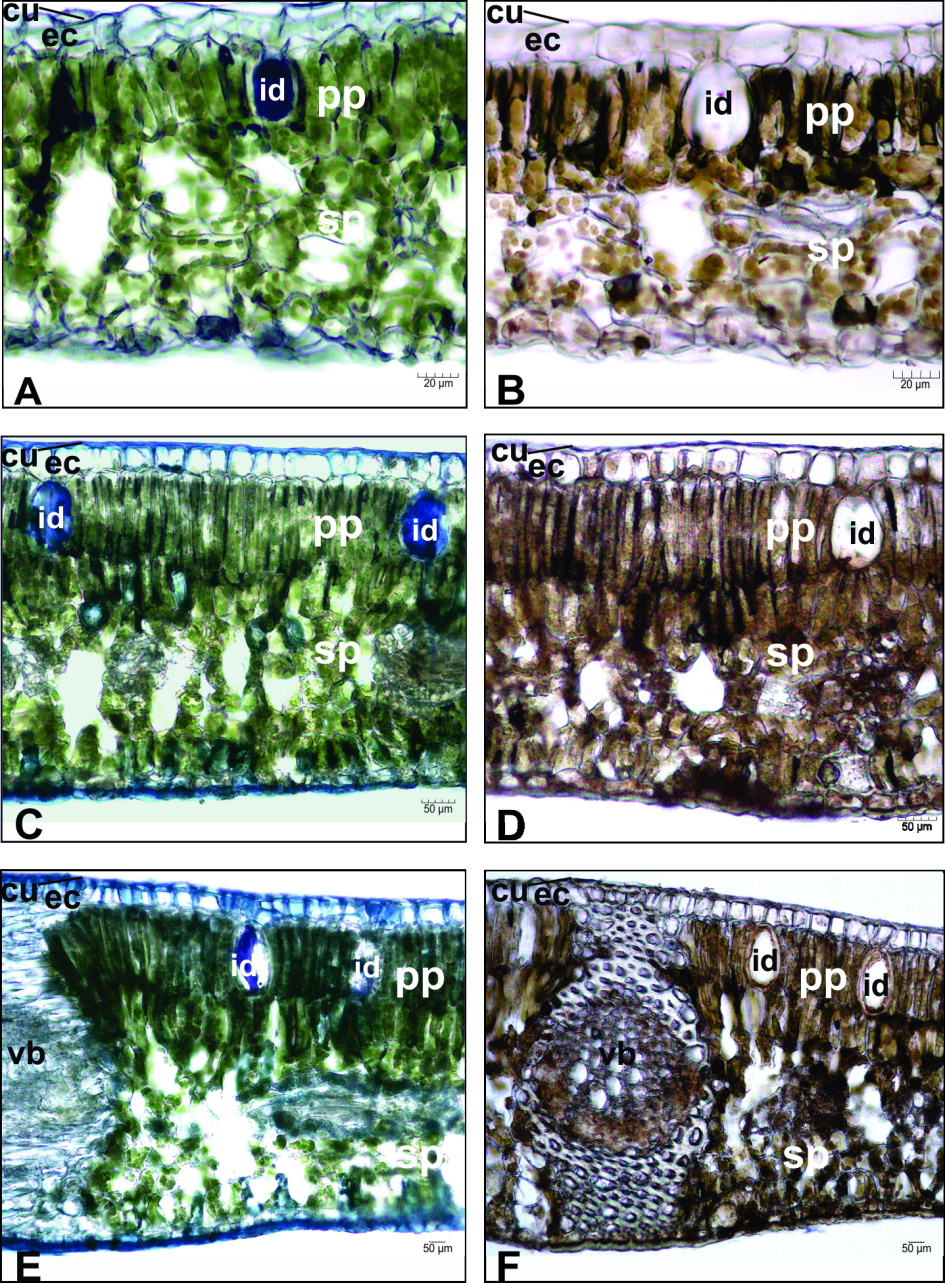
Histochemical analysis of *N*. *megapotamica*. (Sample S6: A and B; Sample C1: C and D; C3: E and F; A/C/E: dye NADI; B/D/F: dye Ferric Chloride; cu: cuticle; ec: epidermal cell; id: idioblast; pp: palisade parenchyma; sp: spongy parenchyma; vb: vascular bundle).

We evaluated different tests to identify the anatomical features and histolocalization of the essential oil from *N*. *megapotamica* with different chemotypes. The histochemical analyses of sample S6 (chemotype A) are presented in Fig 2(A) and 2(B), and chemotype B samples are presented in Fig 2(C) and 2(D) (sample C1) and Fig 2(E) and 2(F) (sample C3). Anatomical differences were not observed between samples from the two chemotypes (specimens from region S (S6) and region C (C1 and C3)).

The presence of essential oils in idioblasts was suggested by the chemical reaction with the dye NADI (Fig 2(A), 2(C) and 2(E)), which was corroborated by the positive response with Sudan IV (S2A Fig), a reactive for lipophilic substances, and Nile blue (S2B Fig), reactive for acidic lipids [23]. The ferric chloride reaction (Fig 2(B), 2(D) and 2(F)) did not stain the contents inside the idioblasts, but was positive for the phenolics in the palisade and spongy parenchyma.

Although previous studies have demonstrated the possible presence of volatile chemotypes in *N*. *megapotamica* [15, 16], no analyses have correlated histochemical and essential oil analyses at the same time for this species. Furthermore, our data indicated that both volatile chemotypes (A and B) were accumulated in idioblasts.

The leaves presented an unstratified epidermis and idioblasts distributed on both sides of the leaf between palisade parenchyma and in the spongy parenchyma (S3 Fig). The samples S6 (chemotype A) and C1 and C3 (chemotype B) presented different amounts of idioblasts. Likewise, all the samples presented a higher number of idioblasts on the adaxial surface than the abaxial surface (p < 0.001). The difference in the number of idioblasts on each side was calculated by the average amount of idioblasts in the same leaf region. This data is shown in S4 Fig.

**Fig 3:**
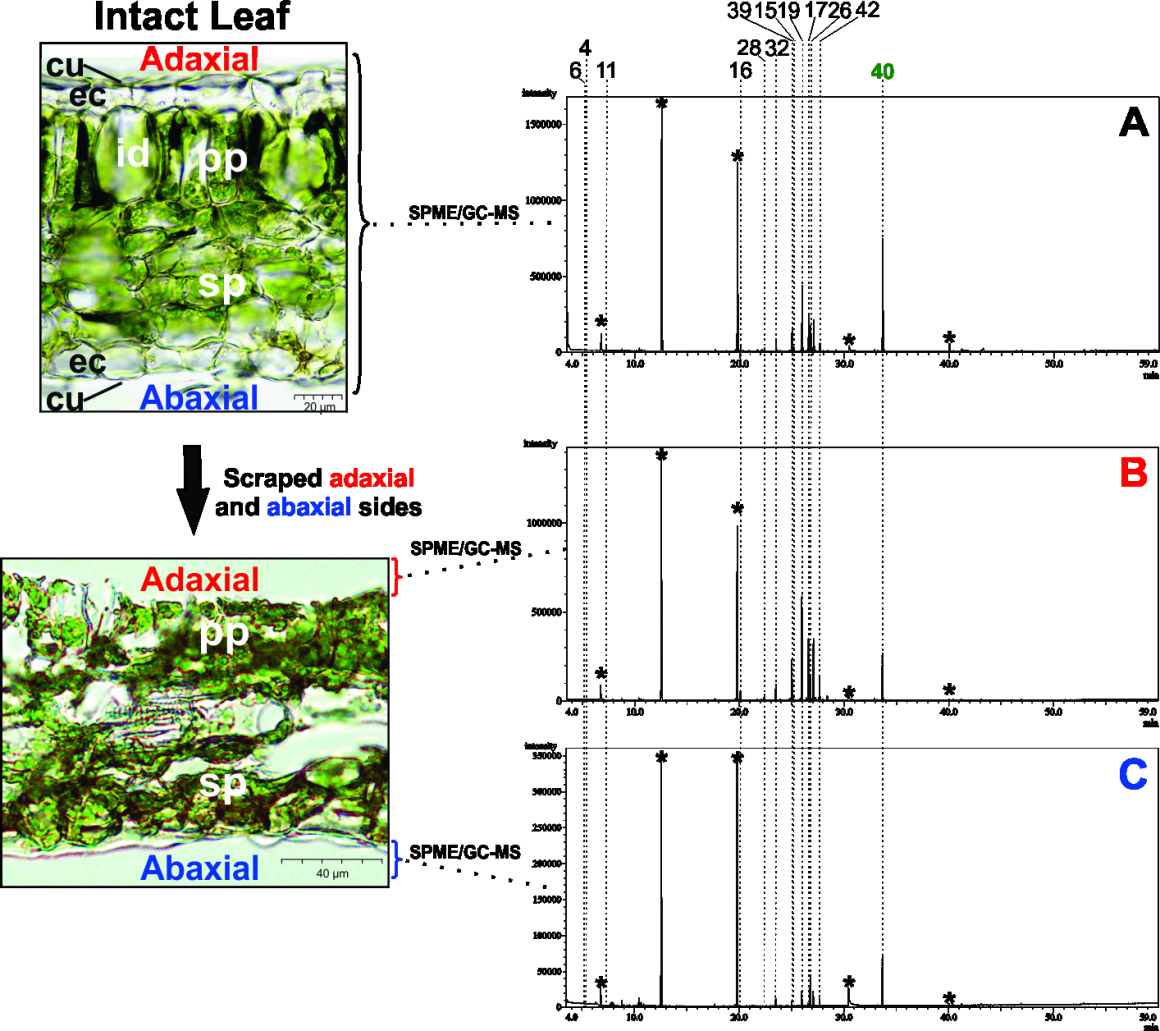
**SPME/GC-MS analysis of intact leaves (A) and scraped adaxial (B) and abaxial (C) surfaces from S6**. (*peak products of SPME fiber; cu: cuticle; ec: epidermal cell; id: idioblast; pp: palisade parenchyma; sp: spongy parenchyma; id: idioblast).

To evaluate the difference between chemical composition on the adaxial and abaxial leaf surfaces, the SPME (solid phase microextraction)/GC-MS was carried out using the samples S6 and S7. The adaxial and abaxial surfaces were scraped and added into vials for extraction. The compounds identified are described in S2 Table, and the chromatograms are illustrated in Fig 3 and S4 Fig, respectively.

The chemical composition of abaxial and adaxial leaf sides were similar for sample S6. The main compounds identified were germacrene D (peak 19) with 17.0% on intact leaves, 26.0% on the abaxial side, 25.3% on the adaxial side, as well as the presence of α-bisabolol (peak 40) with 31.4% on intact leaves, 41.9% on the abaxial side, and 11.6% on the adaxial side.

A second specimen collected from the S region (S7) were included in the analysis, which presented a distinct chemical profile (absence of α-bisabolol and major compound β-bisabolene), with only small differences between abaxial and adaxial sides of the leaf (S2 Table and S5 Fig).

Benedict et al. [28] demonstrated that the formation of β-bisabolene and α-bisabolol has a common precursor, bisabolyl cation, but α-bisabolol suffers solvolysis dependent on Mg^2+^ and β-bisabolene is generated by one deprotonation.

Overall, the data obtained from the histolocalization analyses of *N*. *megapotamica* chemotypes suggested the presence of essential oil in the idioblasts on both leaf surfaces, and the chemotype A accumulated α-bisabolol on both leaf sides. Furthermore, the phenolic compounds were distributed in palisade parenchyma and spongy parenchyma for all the chemotypes.

### Analyses of metabolites by LC-DAD-MS

**Fig 4:**
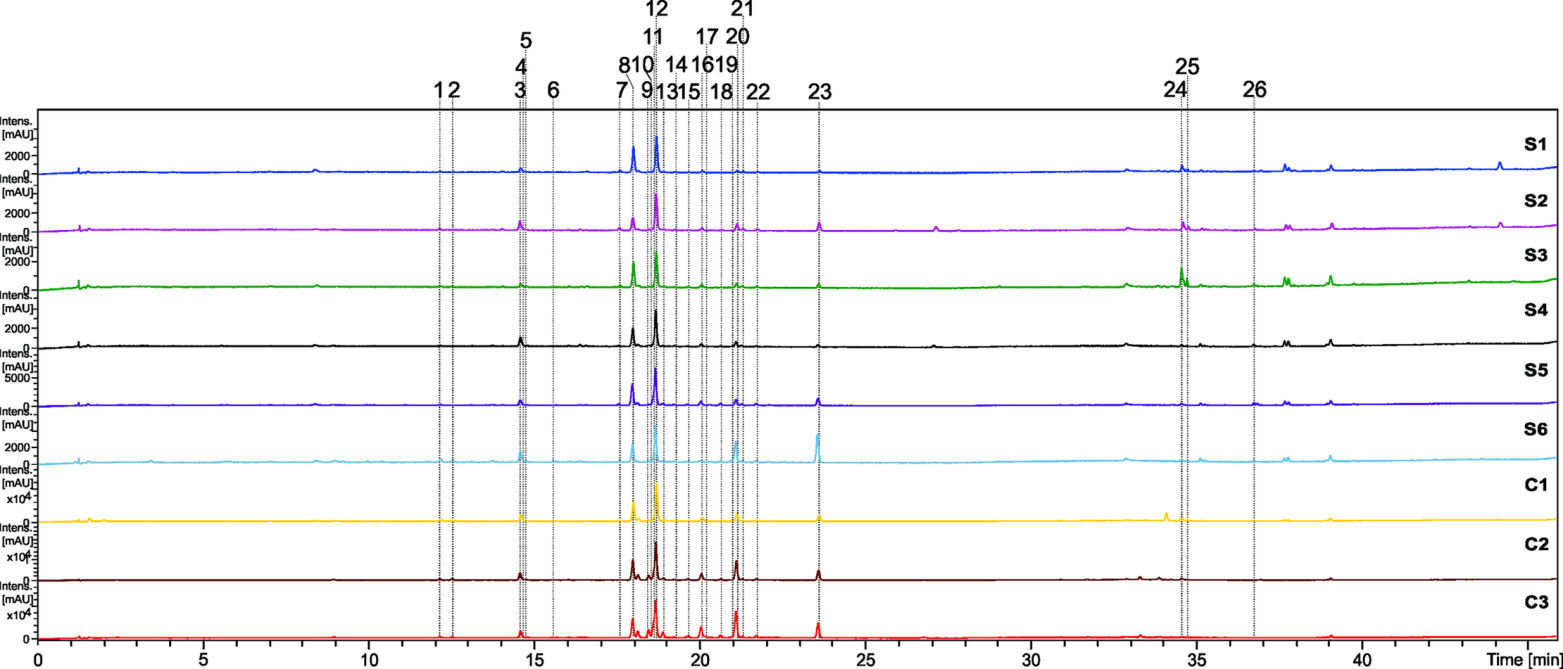
Chromatograms a 245–380 nm of *Nectandra megapotamica* (samples S1-S6 and C1-C3).

The chemical constituents of extracts from *N*. *megapotamica* were evaluated by LC-DAD-MS, and their constituents were identified. The chromatograms and the identifications are shown in Fig 4 and Table 3, respectively.

**Table 3:** Compounds identified from *Nectandra megapotamica* extracts by LC-DAD-MS.

Peak	Compound	RT	Molecular Formula	UV (nm)	[M+H]^+^ *m/z*	[M-H]^-^ *m/z*	MS/MS (negative mode) *m/z*
1	Procyanidin dimer (B type)	12.2	C_30_H_26_O_12_	279	579.1494	577.1327	407, 339, 289, 245, 161
2	Epicatechin^st^	12.5	C_15_H_14_O_6_	279	291.0872	289.0709	245
3	Procyanidin trimer (A type)	14.6	C_45_H_36_O_18_	280	865.1980	863.1785	693, 559, 411, 407, 289, 161
4	Procyanidin trimer (A type)	14.6	C_45_H_36_O_18_	280	865.1973	863.1808	693, 559, 411, 407, 289, 161
5	Procyanidin trimer (B type)	14.8	C_45_H_38_O_18_	280	867.2135	865.1989	577, 451, 407, 289, 261, 217, 161
6	Procyanidin tetramer (B type)	15.5	C_60_H_50_O_24_	280	1155.2768	1153.2641	863, 576, 407, 289
7	Unknown	17.6	C_23_H_21_NO_8_	280, 380	440.1365	438.1123	287
8	Vitexin	18.0	C_21_H_20_O_10_	265, 335	433.1169	431.0972	311, 283, 163
9	Procyanidin dimer (B type)	18.4	C_30_H_26_O_12_	279	579.1308	577.1308	407, 203, 245, 289
10	Quercetin-*O*-hexoside	18.5	C_21_H_20_O_12_	265, 348	465.1060	463.0867	300, 271, 255
11	Quercetin *O*-deoxyhexosyl-hexoside	18.6	C_27_H_30_O_16_	268, 350	611.1607	609.1443	300, 271, 255, 245, 179
12	Isovitexin	18.7	C_21_H_20_O_10_	266, 335	433.1166	431.0971	341, 323, 311, 283
13	Quercetin *O*-hexoside	18.9	C_21_H_20_O_12_	270, 348	465.1018	463.0861	300, 271, 255, 243
14	Luteolin *O-*deoxyhexosyl hexoside	19.3	C_27_H_30_O_15_	270, 340	595.1682	593.1429	285
15	Luteolin *O-*deoxyhexosyl hexoside	19.6	C_27_H_30_O_15_	270, 340	595.1674	593.1427	285
16	Quercetin *O*-pentoside	20.1	C_20_H_18_O_11_	270, 352	435.0755	433.0755	300, 271, 255
17	Quercetin *O*-deoxyhexoside	20.2	C_21_H_20_O_11_	270, 352	449.1076	447.0852	300, 255
18	Luteolin *O*-deoxyhexosyl hexoside	20.7	C_27_H_30_O_15_	266, 340	595.1652	593.1492	285, 225
19	Quercetin *O*-deoxyhexoside	21.0	C_21_H_20_O_11_	270, 352	449.1062	447.0906	300, 271, 255
20	Quercetin *O*-deoxyhexoside	21.1	C_21_H_20_O_11_	270, 352	449.1080	447.0916	300, 271, 255
21	Apigenin *O-*deoxyhexosyl hexoside	21.3	C_27_H_30_O_14_	270, 338	579.1699	577.1531	269
22	Luteolin *O*-pentoside	21.7	C_20_H_18_O_10_	265, 340	419.0984	417.0812	285, 255, 227
23	Luteolin *O-*hexoside	23.6	C_21_H_20_O_10_	263, 340	433.1121	431.0986	285, 255, 227
24	Unknown	34.6	C_39_H_32_O_14_	280, 320	725.1886	723.1734	285, 255, 187, 163
25	Unknown	34.8	C_39_H_32_O_14_	280, 320	725.1872	723.1754	285, 255, 187, 163
26	Unknown	36.8	C_19_H_22_O_3_	284	-	297.1501	183

Mass errors and mSigma below 5 ppm and 30, respectively; RT: retention time, St: confirmed by injection of the authentic standard.

The LC-DAD-MS analysis revealed a high similarity between the samples, unlike the chemical profile found in essential oils. The compounds were identified based on their fragmentation patterns which were compared to the literature, such as proanthocyanidins [29] and glycosylated flavonoids (*O*- and *C*-glycosides) [30, 31].

The peaks **1**, **3**, **4**, **5**, **6,** and **9** were identified as flavan-3-ol derivatives. The main fragments found were yielded from Retro-Diels-Alder (RDA) fragmentation (losses of 152 *u*) followed by dehydration (losses of 170 *u*), as well as the fragments related to catechin/epicatechin in monomeric form [29]. These peaks also showed compatible UV (_λmax_ ≈ 277–280 nm), and were identified as procyanidin dimers B type (peaks **1** and **9**), procyanidin trimer A and B type (peaks **3**, **4** and **5**), and procyanidin tetramer B type (peak **6**). The B- and A-type proanthocyanidins with the same composition of flavan-3-ol units presented mass differences of 2 *u*. The A-type procyanidins were formed by 4→8 C–C bond and an interflavonoid C–O bond, and in B type by 4→8 or 4→6 C–C bonds between flavan-3-ol monomers [32].

The peaks **8** and **12** were identified as vitexin and isovitexin. Vitexin has a shorter elution time compared to isovitexin [33]. The *m*/*z* 311 fragment is characteristic of *C*-glycoside flavonoids, which is yielded from glucose cleavage and loss of C_4_H_8_O_4_ (120 *u*) with subsequent ring contraction and loss of CO (28 *u*) confirmed by the ion *m*/*z* 283. Both compounds also exhibit UV bands at λ_max_ ≈ 280 and 335 nm compatible with flavones [34].

The peaks **10**, **11**, **13**, **17**, **19,** and **20** showed UV bands (λ_max_ ≈ 270 and 350 nm) compatible with flavonols, and in MS/MS aglycone *m*/*z* 300 [Y_0_-H]^-^ as the principal ion fragment produced by the elimination of the glycoside (deoxyhexose and/or hexoside). In addition, the contraction of the ring followed by losses of CO (*m*/*z* 271 [Y_0_-H-CO]^-^) and a water molecule (*m*/*z* 255 [Y_0_-H-CO-H_2_O]^-^) [35]. Thus, the compounds were identified as quercetin *O*-hexoside (peaks **10** and **13**), quercetin *O*-deoxyhexoside (peaks **17**, **19** and **20**), quercetin *O*-deoxyhexosyl-hexoside (peak **11**). The compound **16** showed a loss of 132 *u* corresponding to a pentose and UV characteristic of flavonol (λ_max_ ≈ 270 and 352 nm), thus, was identified as quercetin *O*-pentoside [36].

Peaks **14**, **15**, **18**, **21**, **22,** and **23** were derived from luteolin and exhibit UV spectra characteristic of flavones (λ_max_ ≈ 280 and 340 nm). The ion at *m*/*z* 285 was observed and yielded from losses of 308 *u* (deoxyhexose + hexose) for peaks **14**, **15,** and **18**, characterizing these compounds as luteolin *O*-deoxyhexosyl hexoside [37]. The peaks **23** and **22** showed losses of 162 *u* (hexoside) and 132 *u*, suggesting the compounds luteolin *O*-hexoside and luteolin *O*-pentoside, respectively [38, 39]. The peak **21** was characterized as apigenin *O*-deoxyhexosyl hexoside because its fragmentation showed the fragment ion at *m*/*z* 269 (aglycone) yielded from the loss of 308 *u* (deoxyhexose + hexose).

The only previous report of phenolic compounds from *N*. *megapotamica* was described by Garcez et al. [40], who described epicatechin. In the *Nectandra* genus, epicatechin was reported for *N*. *amazonum* [41], *N*. *cissiflora* [42], vitexin, isovitexin, and epicatechin in *Nectandra cuspidata* [43] and quercetin-3-*O*-rhamnoside in *Nectandra grandiflora* [44]. Thus, the other compounds of the *Nectandra* genus identified herein (peak **1, 3, 4, 5, 6, 7, 9, 10, 11, 13, 14, 15, 16, 18, 21, 22** and **23**) are described for the first time, demonstrating the capacity of *N*. *megapotamica* to accumulate other *O-*glycosylated flavones and flavonols, as well as proanthocyanidins.

In the present study, we observed that the secondary metabolism of medium to high polarity was highly conserved, even though *N*. *megapotamica* produced different volatile chemotypes. These compounds may remain unchanged due to basal actions that are exerted, acting as fundamental compounds for protection against ultraviolet, antimicrobial activity, response to biotic and abiotic stress, and protection against oxidative stress [45, 46]. The maintenance of this class of compounds may be vital for their survival.

A similar study was done using another Lauraceae species, *Cryptocarya mandioccana* Meisner, which presented a similar chemical profile for flavonoid glycosides, while the styrylpyrones presented high variability and were classified into four chemotypes. The authors attributed the production of such chemotypes to genetic or environmental influences (mainly soil and climate) [17].

### Chiral analyses of α-bisabolol from *N*. *megapotamica*

α-Bisabolol is a monocyclic sesquiterpene that has two chiral centers (C1 and C7), and is found in nature in the following four isomers: (+) and (-)-α-bisabolol and (+) and (-)-epi-α-bisabolol (structures are presented in the S6 Fig). The stereochemistry of bisabolol in *N*. *megapotamica* has not yet been described. Such stereochemistry is important since there are high contents of bisabolol in leaves of *N*. *megapotamica* with chemotype A (almost 94%), and could be an important source of this compound for commercial use.

**Fig 5:**
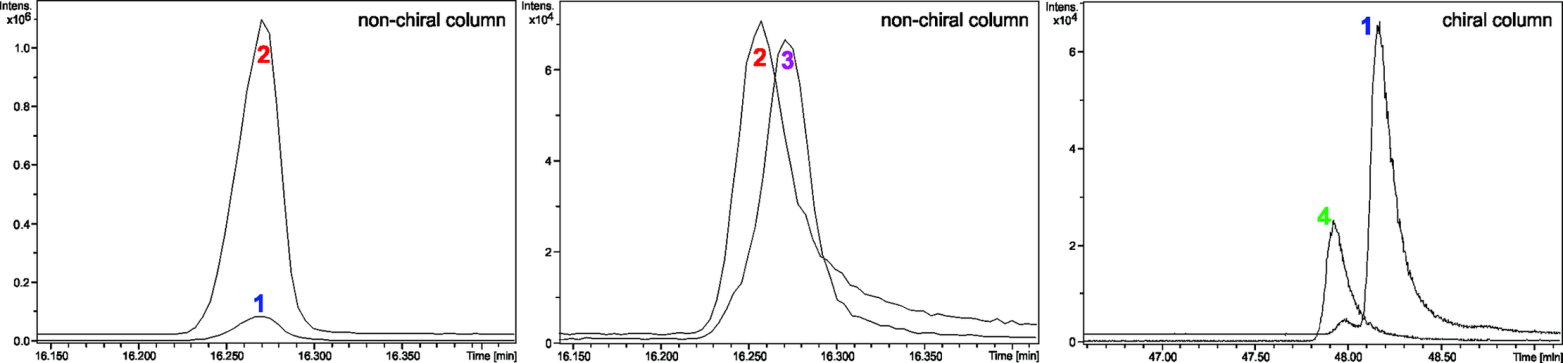
Stereochemical analysis of α-bisabolol present in *Nectandra megapotamica*. 1: compound of *N*. *megapotamica*; 2: α-bisabolol; 3: epi-α-bisabolol; 4: (-)-α-bisabolol.

We analyzed the essential oil from *N*. *megapotamica* and compared samples with α-bisabolol and epi-α-bisabolol by non-chiral column and compared (-)-α-bisabolol by chiral column (Fig 5). The sesquiterpenes α-bisabolol and epi-α-bisabolol are diastereomers, thus, they showed different physio-chemical properties and different retention times in non-chiral columns. When comparing the α-bisabolol present in *N*. *megapotamica* to α-bisabolol (Fig 5 - compounds 1 and 2), we observed an overlap of the peaks in a non-chiral column (ZB-5MS). In the second analysis in the non-chiral column, α-bisabolol and epi-α-bisabolol (Fig 5 - compounds 2 and 3) revealed different retention times of peaks from alpha and epi isomers, and demonstrated that the chromatographic method was efficient to separate these diastereomers. However, the bisabolol present in *Nectandra* is α-bisabolol.

Similarly, the samples were also analyzed by a chiral column. We detected an evident change in retention time comparing the α-bisabolol from *N*. *megapotamica* and (-)-α-bisabolol (Fig 5 - compounds 1 and 4) from *E*. *erythropappus*, the plant that is currently used to obtain the isoform (-)-α-bisabolol. Therefore, we conclude that α-bisabolol present in *N*. *megapotamica* is the compound (+)-α-bisabolol, since it presented a different retention time from (-)-α-bisabolol in a chiral column.

The methods used for stereochemical identification are based on previously described analyses, such as Son et al [47] and Attia, Kim and Ro [48]. These studies reported identification of (-)-α-bisabolol and (+)-epi-α-bisabolol by the chromatographic analyses in non-chiral column (such as DB1-MS column), and subsequent chiral column analysis (α-cyclodextrin chiral) by comparing standards of molecules with known stereochemistry.

The presence of α-bisabolol in *N*. *megapotamica* from the S region has been reported [15], however, has not been stereochemicaly characterized, as reported herein.

The (+)-α-bisabolol has been reported in the extracts of *Artemisa annua* [49], *Cymbopogon flexuosus* [50], and *Populus balsamifera* [51]. Some biological activities have been described in the literature, as the high cytotoxic activity in human glycoma cells (U251) [51]. The combination of the amino acid arginine and (+/-)-α-bisabolol has been reported as a useful formulation for the treatment and prevention of dental cavities [52]. In addition, ecological studies have demonstrated the antifeedant actions of balsam poplar *Populus balsamifera*, which is rich in (+)-α-bisabolol, against rodents (*Lepus americanus*) [53].

Therefore, according to our findings, *N*. *megapotamica* is a rich source of (+)-α-bisabolol with production up to 93.7% (Table 2) in the renewable resource (leaves), which demonstrates this species potential in the development of technological products.

## Conclusion

The volatile compounds of *N*. *megapotamica* from different geographic locations differed in chemotypes, while for other classes, such as phenolics, composition was highly preserved. Such findings could be related to the crucial physiological functions of phenolic compounds, which help the species to survive. The differentiation of volatiles may be associated with some genetic gap and local environmental influences.

The stereochemical determination of (+)-α-bisabolol shows that *N*. *megapotamica* has high potential for further exploration. *N*. *megapotamica* can be used in future studies to better understand the effects of genetic and environmental factors on chemotypic expression.

## Supporting information

S1 Table Identification and location of the studied material. (PDF)

S2 Table Compounds identified by CG-MS of S6 and S7 after scraping method. The individuals S6 and S7 were identified as *Nectandra megapotamica*. S6A: analysis of adaxial surface; S6B: analysis of abaxial surface; S6IP: analysis of intact leaves; S7A: analysis of adaxial surface; S7B: analysis of abaxial surface; S7IP: analysis of intact leaves; n: number; RI: retention index of compound; RIL: retention index of literature.
(PDF)

S1 Fig Geographic distribution of *Nectandra megapotamica* analysed in the study. (MS: State of Mato Grosso do Sul; SP; State of São Paulo; C1-C3: Samples from Campo Grande city; M1: Samples from Maracajú city; P1 and P2: Samples from Ponta Porã city; S1-S7: Samples from São Paulo city.
(PDF)

S2 Fig Histochemical analysis of S7 leaves. (A: dye Sudan IV; B: dye Nile Blue; cu: cuticle; ec: epidermal cell; id: idioblast; pp: palisade parenchyma; sp: spongy parenchma).
(PDF)

S3 Fig Idioblasts present on the adaxial and abaxial surfaces of S7 leaves. (dye: NADI; cu: cuticle; ec: epidermal cell; id: idioblast; pp: palisade parenchyma; sp: spongy parenchma).
(PDF)

S4 Fig Number of idioblasts between the adaxial and abaxial surfaces of *Nectandra megapotamica*. Sample S6: Chemotype A; Sample C1 and C3: Chemotype B.
(PDF)

S5 Fig SPME analysis of intact leaves and adaxial and abaxial surfaces of S7 by GC-MS. (*peak products of SPME fiber; cu: cuticle; ec: epidermal cell; id: idioblast; pp: palisade parenchyma; sp: spongy parenchyma; id: idioblast).
(PDF)

S6 Fig Four different configurations of α-bisabolol. (PDF)
