# Supplementary material for: In depth investigation of the metabolism of Nectandra megapotamica chemotypes
Source: PLoS One. 2018 Aug 6;13(8):e0201996. doi: 10.1371/journal.pone.0201996 (PMC6078319; doi:10.1371/journal.pone.0201996)
Supplement: S1 Table — (PDF) [file pone.0201996.s001.pdf]

S1 Table: Identification and location of the studied material.

Sample	Material	Location<br>city-state-country (abv)	Day of collect	Vouchers
C1	*N. megapotamica*	Campo Grande - MS, Brazil (C)	17/10/2013	CGMS 46207
C2	*N. megapotamica*	Campo Grande - MS, Brazil (C)	19/11/2013	CGMS 66304
C3	*N. megapotamica*	Campo Grande - MS, Brazil (C)	19/11/2013	CGMS 66303
M1	*N. megapotamica*	Maracaju - MS, Brazil (M)	05/03/2014	CGMS 66314
P1	*N. megapotamica*	Ponta Porã - MS, Brazil (P)	05/03/2014	CGMS 66312
P2	*N. megapotamica*	Ponta Porã - MS, Brazil (P)	05/03/2014	CGMS 66313
S1	*N. megapotamica*	São Paulo - SP, Brazil (S)	01/11/2014	SPSF 39648
S2	*N. megapotamica*	São Paulo - SP, Brazil (S)	01/11/2014	SPSF 39648
S3	*N. megapotamica*	São Paulo - SP, Brazil (S)	01/11/2014	SPSF 39648
S4	*N. megapotamica*	São Paulo - SP, Brazil (S)	01/11/2014	SPSF 39648
S5	*N. megapotamica*	São Paulo - SP, Brazil (S)	01/11/2014	SPSF 39648
S6	*N. megapotamica* - seedling	São Paulo - SP, Brazil (S)	01/11/2014	SPSF 39648
S7	*N. megapotamica* - seedling	São Paulo - SP, Brazil (S)	01/11/2014	SPSF 39648

Abv: abbreviation
