# Supplementary material for: In depth investigation of the metabolism of Nectandra megapotamica chemotypes
Source: PLoS One. 2018 Aug 6;13(8):e0201996. doi: 10.1371/journal.pone.0201996 (PMC6078319; doi:10.1371/journal.pone.0201996)
Supplement: S2 Table — The individuals S6 and S7 were identified as Nectandra megapotamica. S6A: analysis of adaxial surface; S6B: analysis of abaxial surface; S6IP: analysis of intact leaves; S7A: analysis of adaxial surface; S7B: analysis of abaxial surface; S7IP: analysis of intact leaves; n: number; RI: retention index of compound; RIL: retention index of literature. (PDF) [file pone.0201996.s002.pdf]

S2 Table. Compounds identified by CG-MS of S6 and S7 after scraping method.

	Compounds			S6IP	S6A	S6B	S7IP	S7A	S7B
n.	**Monoterpene hydrocarbons**	RI	RIL						
1	camphene	951	954	-	-	-	0.1	-	-
2	limonene	1031	1029	-	-	-	0.3	0.1	-
3	α-phellandrene	1006	1003	-	-	-	0.2	-	-
4	α-pinene	979	939	-	tr	-	0.9	-	-
5	α-terpinolene	1088	1089	-	-	-	0.5	tr	-
6	α-thujene	931	930	tr	-	-	0.1	0.1	-
7	β-myrcene	991	991	-	-	-	0.5	tr	-
8	β-phellandrene	1062	1030	-	-	-	tr	-	-
9	β-pinene	957	979	-	-	-	0.3	0.1	-
10	γ-terpinene	1059	1060	-	-	-	0.1	-	-
11	δ-3-carene	1012	1031	tr	-	-	3.9	0.1	-
12	(E)-β-ocimeno	1040	1050	-	-	-	0.1	-	-
13	(Z)-β-ocimeno	1050	1037	-	-	-	0.8	tr	-
	**Oxygenated Monoterpene**								
14	eucalyptol	1034	1033	-	-	-	0.4	0.2	-
	**Sesquiterpene hydrocarbons**								
15	alloaromadendrene	1464	1486	0.8	1.2	-	1.0	1.0	0.6
16	bicycloelemene	1341	1336	1.4	2.0	-	0.8	0.9	0.7
17	bicyclogermacrene	1499	1500	9.1	15.2	tr	4.9	5.7	4.1
18	cadina-3,5-diene	1454	1454	-	-	-	0.4	0.4	0.3
19	germacrene D	1484	1485	17.0	25.3	26.0	5.8	6.6	6.2
20	sesquisabinene	1461	1460	-	-	-	21.4	20.2	19.1
21	sesquithujene	1408	1417	-	-	-	2.7	1.9	1.3
22	α-copaene	1379	1377	-	-	-	0.3	0.2	0.1
23	α-humulene	1457	1455	-	-	-	0.2	0.2	0.2
24	α-muurolene	1503	1500	-	-	-	0.5	0.6	0.6
25	β-bourbonene	1387	1388	-	-	-	tr	tr	-
26	β-bisabolene	1514	1506	7.5	0.5	-	25.1	29.2	32.8
27	β-curcumene	1515	1516	-	-	-	0.2	0.2	0.3
28	β-elemene	1394	1391	0.7	0.9	-	0.2	0.2	0.2
29	β-sesquiphellandrene	1527	1523	-	-	-	2.2	2.9	3
30	γ-amorphene	1494	1496	-	-	-	0.4	0.4	0.4
31	cis-α-bergamotene	1418	1413	-	-	-	2.0	1.5	0.9
32	trans-caryophyllene	1422	1419	2.4	3.4	tr	1.0	0.8	0.9
33	trans-α-bergamotene	1439	1435	-	-	-	1.0	0.8	0.5
34	Epi-β-santalene	1451	1447	-	-	-	0.9	0.8	0.5
35	(E)-α-bisabolene	1547	1540	-	-	-	3.8	-	6.2
36	(E)-β-farnesene	1487	1457	-	-	-	0.9	1.0	0.8
37	(E)-γ-bisabolene	1536	1531	-	-	-	12.3	-	17.9
38	(Z)-α-bisabolene	1505	1507	-	-	-	0.5	0.7	0.8
39	(Z)-β-farnesene	1461	1446	5.8	9.3	-	-	-	-
	**Oxygenated sesquiterpene**								
**40**	**α-bisabolol**	1687	1686	**31.4**	**11.6**	**41.9**	-	-	-

The individuals S6 and S7 were identified as *Nectandra megapotamica*. S6A: analysis of adaxial surface; S6B: analysis of abaxial surface; S6IP: analysis of intact leaves; S7A: analysis of adaxial surface; S7B: analysis of abaxial surface; S7IP: analysis of intact leaves; n: number; RI: retention index of compound; RIL: retention index of literature.
