# Supplementary material for: In depth investigation of the metabolism of Nectandra megapotamica chemotypes
Source: PLoS One. 2018 Aug 6;13(8):e0201996. doi: 10.1371/journal.pone.0201996 (PMC6078319; doi:10.1371/journal.pone.0201996)
Supplement: S1 Fig — (MS: State of Mato Grosso do Sul; SP; State of São Paulo; C1-C3: Samples from Campo Grande city; M1: Samples from Maracajú city; P1 and P2: Samples from Ponta Porã city; S1-S7: Samples from São Paulo city. (PDF) [file pone.0201996.s003.pdf]

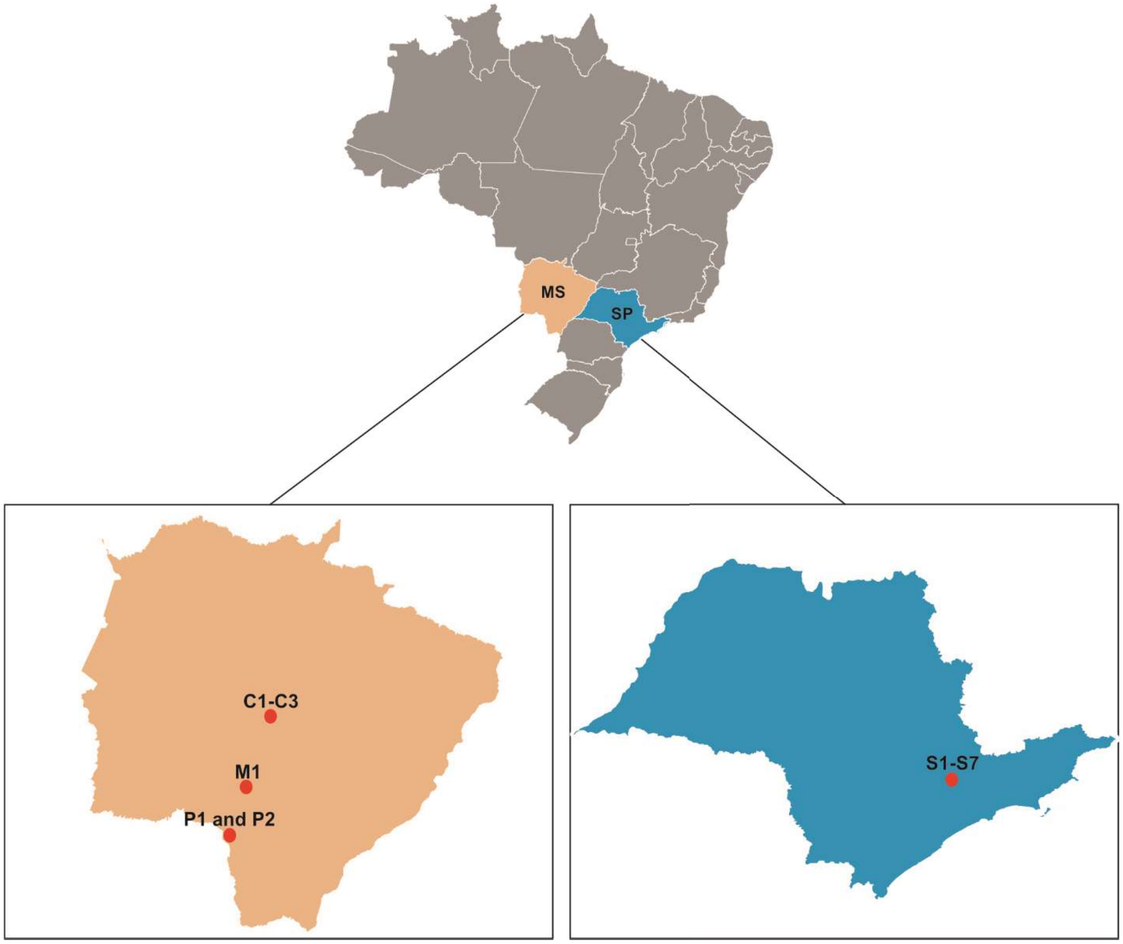

S1 Fig. Geographic distribution of *Nectandra megapotamica* analysed in the study. (MS: State of Mato Grosso do Sul; SP; State of São Paulo; C1-C3: Samples from Campo Grande city; M1: Samples from Maracajú city; P1 and P2: Samples from Ponta Porã city; S1-S7: Samples from São Paulo city.
