# Supplementary material for: In depth investigation of the metabolism of Nectandra megapotamica chemotypes
Source: PLoS One. 2018 Aug 6;13(8):e0201996. doi: 10.1371/journal.pone.0201996 (PMC6078319; doi:10.1371/journal.pone.0201996)
Supplement: S2 Fig — (A: dye Sudan IV; B: dye Nile Blue; cu: cuticle; ec: epidermal cell; id: idioblast; pp: palisade parenchyma; sp: spongy parenchma). (PDF) [file pone.0201996.s004.pdf]

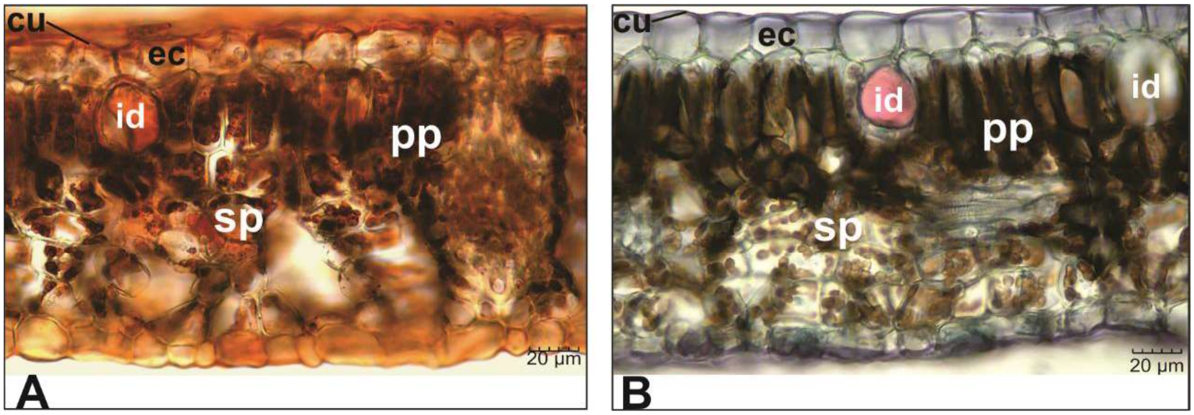

S2 Fig. Histochemical analysis of S7 leaves. (A: dye Sudan IV; B: dye Nile Blue; cu: cuticle; ec: epidermal cell; id: idioblast; pp: palisade parenchyma; sp: spongy parenchma).
