# Supplementary material for: In depth investigation of the metabolism of Nectandra megapotamica chemotypes
Source: PLoS One. 2018 Aug 6;13(8):e0201996. doi: 10.1371/journal.pone.0201996 (PMC6078319; doi:10.1371/journal.pone.0201996)
Supplement: S3 Fig — (dye: NADI; cu: cuticle; ec: epidermal cell; id: idioblast; pp: palisade parenchyma; sp: spongy parenchma). (PDF) [file pone.0201996.s005.pdf]

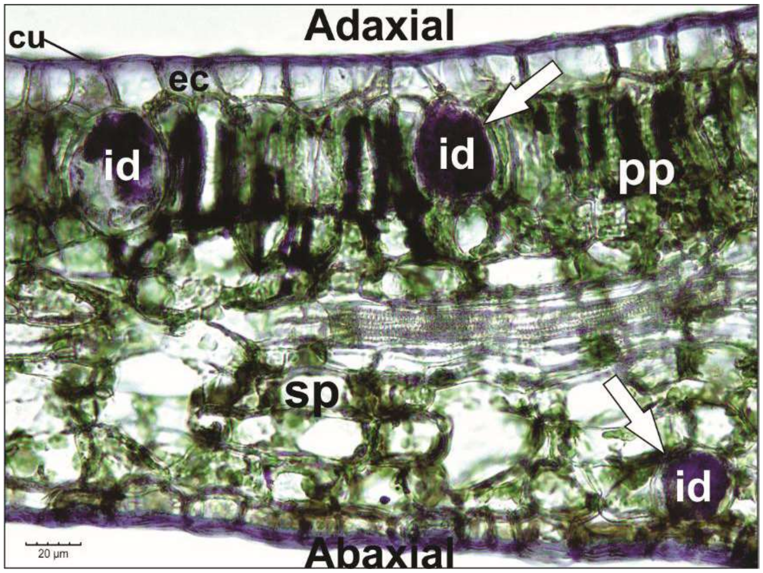


**S3 Fig. Idioblasts present on the adaxial and abaxial surfaces of S7 leaves**. (dye: NADI; cu: cuticle; ec: epidermal cell; id: idioblast; pp: palisade parenchyma; sp: spongy parenchma).
