# Supplementary material for: In depth investigation of the metabolism of Nectandra megapotamica chemotypes
Source: PLoS One. 2018 Aug 6;13(8):e0201996. doi: 10.1371/journal.pone.0201996 (PMC6078319; doi:10.1371/journal.pone.0201996)
Supplement: S4 Fig — Sample S6: Chemotype A; Sample C1 and C3: Chemotype B. (PDF) [file pone.0201996.s006.pdf]

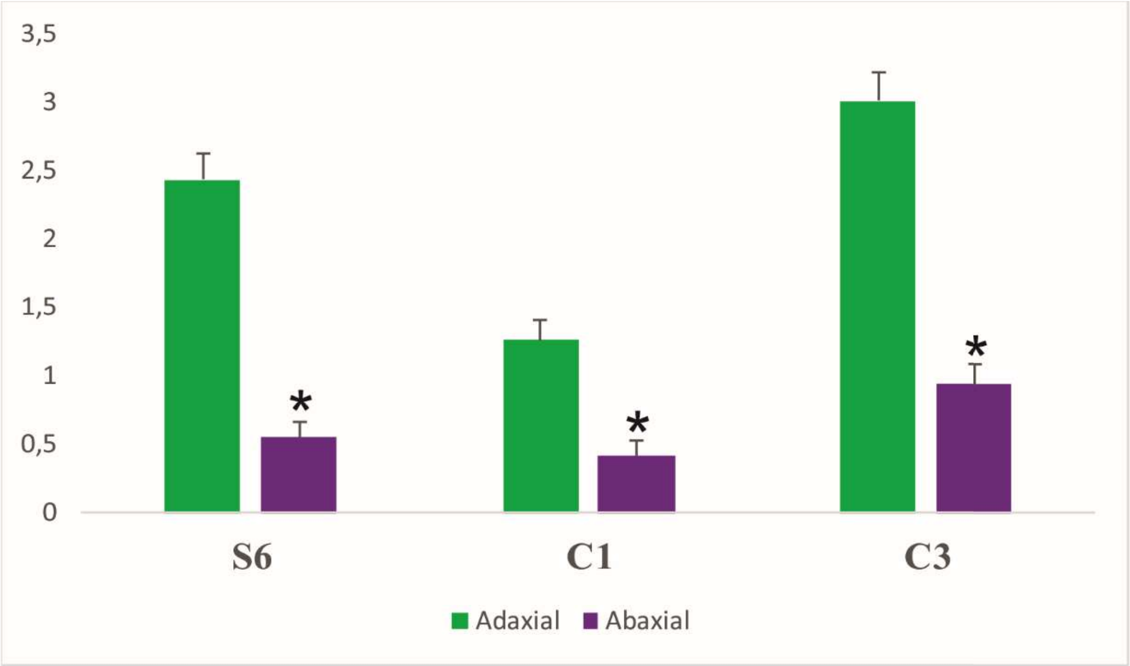

S4 Fig. Number of idioblasts between the Adaxial and Abaxial surfaces of *Nectandra megapotamica*. Sample S6: Chemotype A; Sample C1 and C3: Chemotype B.
