# Supplementary material for: In depth investigation of the metabolism of Nectandra megapotamica chemotypes
Source: PLoS One. 2018 Aug 6;13(8):e0201996. doi: 10.1371/journal.pone.0201996 (PMC6078319; doi:10.1371/journal.pone.0201996)
Supplement: S5 Fig — (*peak products of SPME fiber; cu: cuticle; ec: epidermal cell; id: idioblast; pp: palisade parenchyma; sp: spongy parenchyma; id: idioblast). (PDF) [file pone.0201996.s007.pdf]

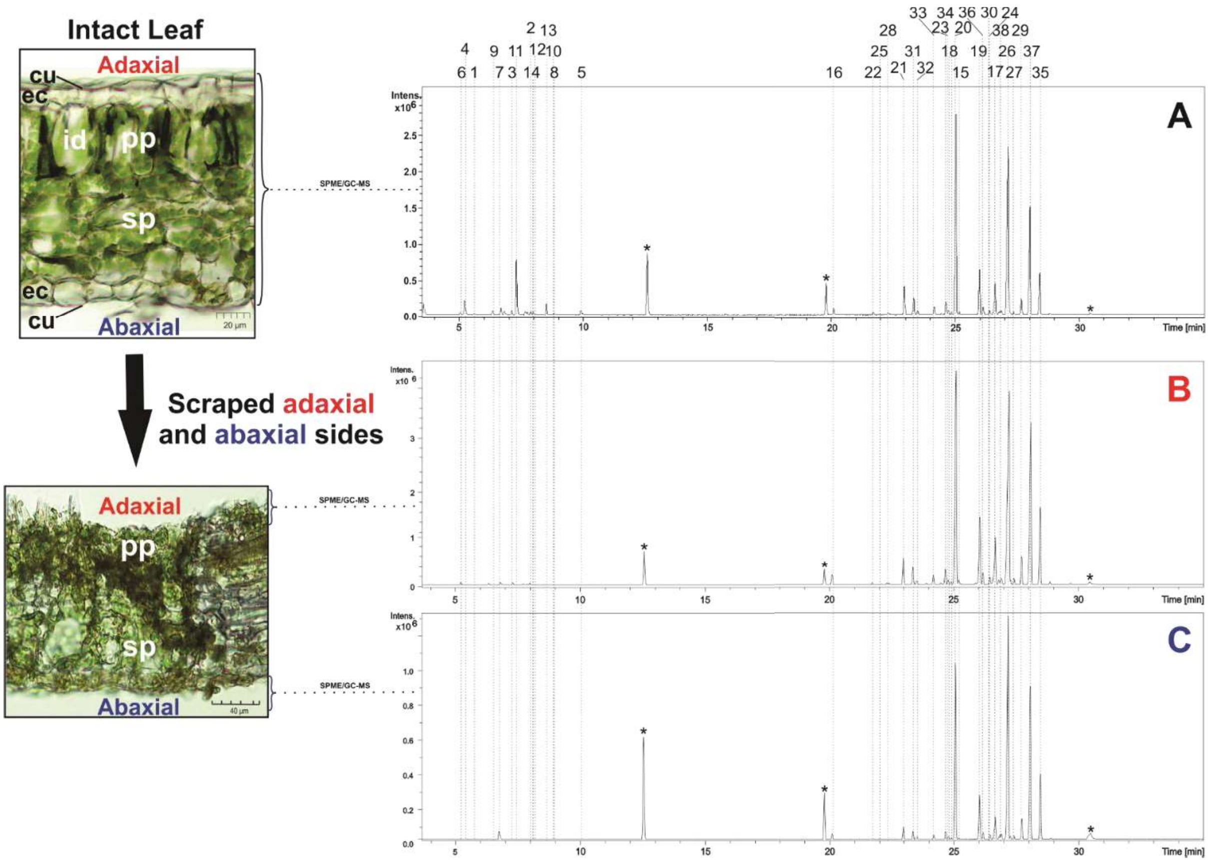

S5 Fig. SPME analysis of intact leaves and adaxial and abaxial surfaces of S7 by GC-MS. (*peak products of SPME fiber; cu: cuticle; ec: epidermal cell; id: idioblast; pp: palisade parenchyma; sp: spongy parenchyma; id: idioblast).
