# Supplementary figures and images for: In depth investigation of the metabolism of Nectandra megapotamica chemotypes
Source: PLoS One. 2018 Aug 6;13(8):e0201996. doi: 10.1371/journal.pone.0201996 (PMC6078319; doi:10.1371/journal.pone.0201996)

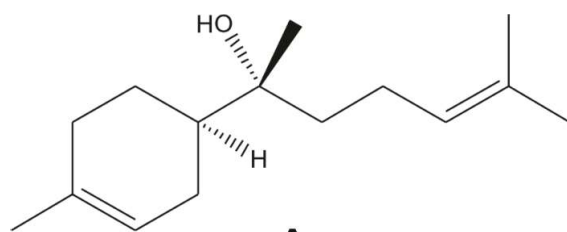

**A**

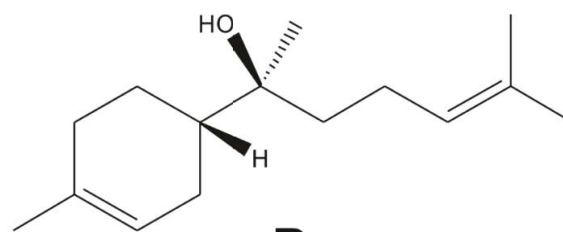

**B**

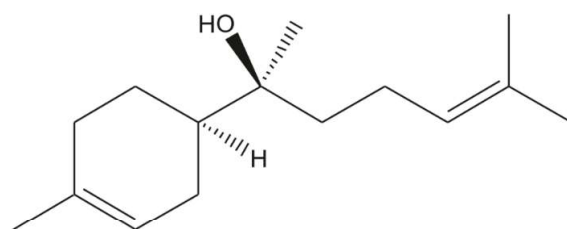

**C**

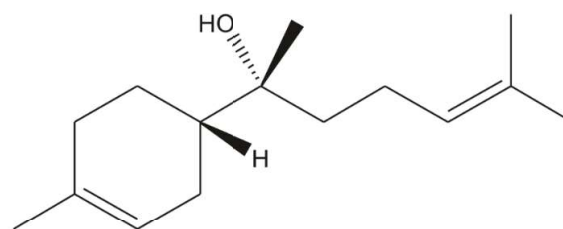

**D**

**S6 Fig. Four different configurations of  $\alpha$ -bisabolol.**

Supplement: S6 Fig — (PDF) [file pone.0201996.s008.pdf]
